# Refractory hypercalcemia due to an ectopic mediastinal parathyroid gland in a hemodialysis patient: a case report

**DOI:** 10.1186/s12882-019-1363-5

**Published:** 2019-05-14

**Authors:** Yingjing Shen, Peipei Fei

**Affiliations:** Department of Nephrology, Third Affiliated Hospital of Second Military Medical University, Shanghai, China

**Keywords:** Hypercalcemia crisis, Hyperparathyroidism, Ectopic parathyroid carcinoma, Hemodialysis

## Abstract

**Background:**

Hypercalcemia crisis is a complex disorder rarely induced by tertiary hyperparathyroidism, which clinically presents as nonsuppressible parathyroid hyperplasia with persistent increased PTH levels and hypercalcemia. It is one of the major risk factors of morbidity and mortality in end-stage renal disease. Parathyroidectomy should be in consideration in dialysis patients with severe hyperparathyroidism who are refractory to medical therapy. The implications and consequences of it, however, are not fully understood.

**Case presentation:**

We present a case of a 70 year-old man disturbed by gastrointestinal manifestations due to hypercalcaemic crisis. The patient had longstanding hypercalcaemia and hyperparathyrodism refractory to calcimimetics, calcitonin, hormone and haemodialysis. A ectopic parathyroid gland in anterior mediastinum was found and elucidated by Tc-99 m scan.

Futhermore, a video-assisted thoracoscopic parathyroidectomy was performed. Histologically, the tumour consisted of densely arranged chief cells immunohistochemically positive for PTH antigens. Consequently, calcium and parathormone were declining stably without any complications.

**Conclusions:**

On account of refractory hypercalcemia and hyperparathyroidism, radionuclide scanning is useful in the diagnosis of ectopic parathyroid gland. it is of great significance for multidisciplinary therapy combing anesthesia, surgical, endocrinology and nephrology staff.

## Background

Hypercalcemic crisis is a rare but life-threatening complication, which is currently defined as a syndrome characterized by a serum calcium level over 3.5 mmol/l(14 mg/dl). Secondary hyperparathyroidism (HPT) is a common clinical issue among hemodialysis patients. For an equivalent amount of parathyroid tissue, a higher secretion rate of parathyroid hormone (PTH) will lead to lower expression of the calcium-sensing receptor (CaSR) expression [1]. As a result, the parathyroid gland is progressively more resistant to vitamin D analogs treatment [2]. Thereafter, tertiary HPT may be developed, which requires prompt diagnosis and rapid treatment to avoid a lethal course. Under such circumstances, parathyroidectomy (PTx) can markedly reduce the serum calcium and PTH immediately.

## Case presentation

A 70-year-old Chinese man was presented in the study. The patient had the past medical history of diabetes, hypertension and cerebral infarction, and had received hemodialysis three times a week due to renal failure for 2 years. SHPT was confirmed in December 2016 since the serum PTH had elevated 100.3 pmol/l(normal range 1.6–6.9 pmol/l). Cinacalcet(at a dose of 25 mg per day) was prescribed based on the concurrent hypercalcemia and hyperphosphatemia. In April 2017, the PTH level was stable at 94.44 pmol/l due to his poor compliance, with normal calcium level(2.06 mmol/). Subsequently, the patient was given calcitriol pulse therapy(at a dose of 2μg for trice weekly) combined with low calcium dialysate(Ca^2+^, 1.25 mmol/l). Unfortunately, the PTH level bounced high up to 197.3 pmol/l dramatically. Since August 2017, the patient was constantly afflicted with the uncomfortable and irritating sensation in the legs urging him to walk or move his legs. Even worse, the patient had more complaints, such as loss of appetite, nausea, constipation, refractory hypertension, generalized weakness and sleep deprivation. Otherwise, he had an normal physical examination. Laboratory test revealed that serum albumin corrected calcium jumped up to 3.96 mmol/l and the alkaline phosphatase(ALP) level was 160u/l(normal range 45-125u/l), supporting the diagnosis of hypercalcemic crisis and tertiary HPT.

Moreover, several examinations were also completed to identify any possibilities. However, the patient had no bone pain or facture, and no abnormality was revealed in bone X-ray imaging. Besides, M protein and tumor markers were negative as well. Surprisingly, no abnormality was found in parathyroid ultrasound around neck. Given the threat of hypercalcemia crises, continuous renal replacement therapy(CRRT) with low calcium dialysate(Ca^2+^ 1.25 mmol/l) was applied instantly. Meanwhile, calcitriol pulse therapy was ceased while higher-dose cinacalcet(50 mg per day) was reused again. In addition, calcitonin was subcutaneously injected at a dose of 50u per day to lower the blood calcium, but poor response was achieved, and the serum albumin corrected calcium kept elevating to 3.77 mmol/l. Therefore, hormone therapy(intravenous injection of 40 mg methylprednisolone for 7 day, and oral administration of 20 mg prednisone until operation) was considered.

**Fig. 1:**
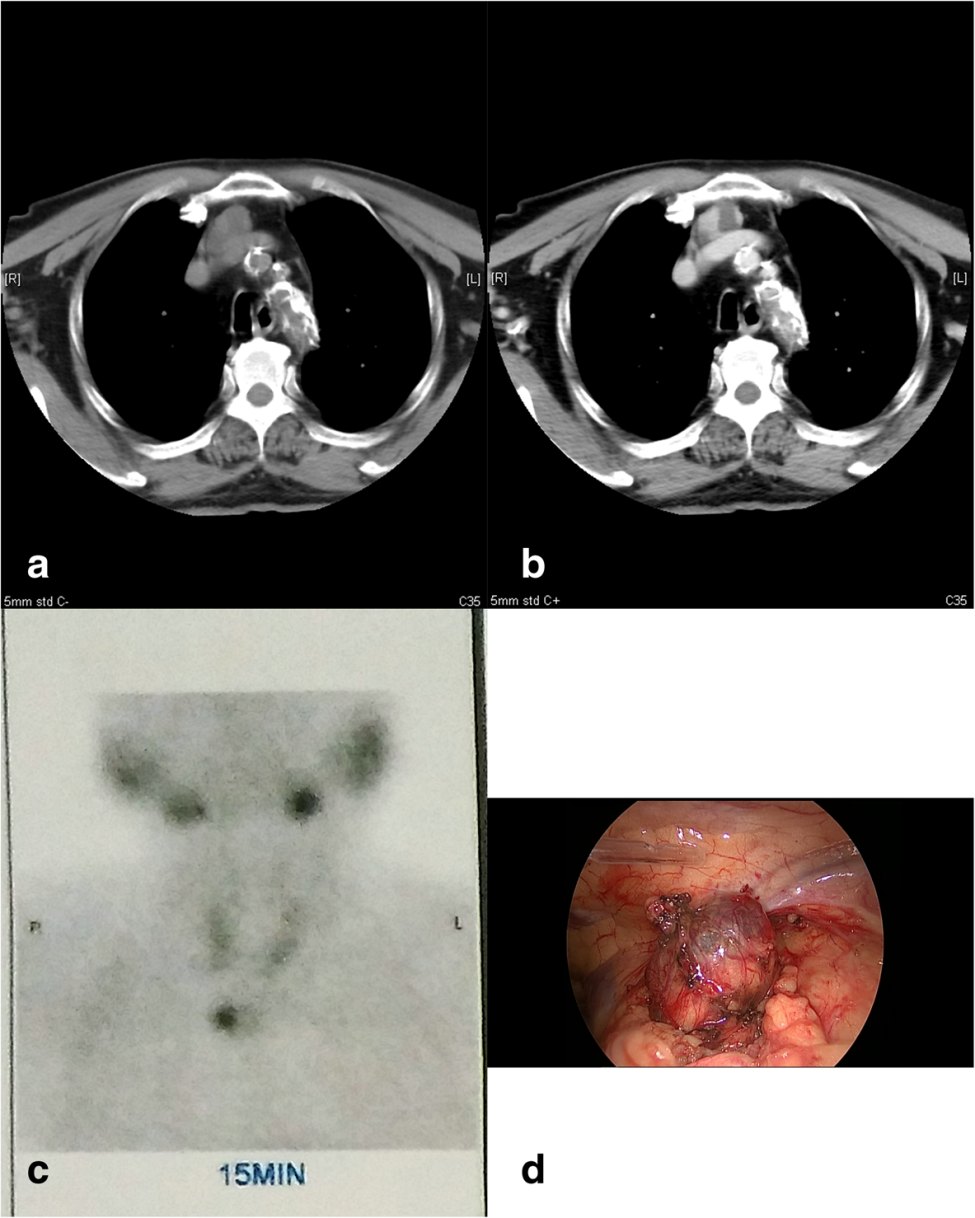
**a** A neck CT scan had revealed a 2.5*1.9 cm mass(arrow) in the upper region of anterior mediastinum; **b** A contrast-enhanced neck CT scan had displayed nodular mixed density; **c** Tc-99 m dual-phases parathyroid scan had detected a hyperfunctioning parathyroid gland(arrow) in the upper region of anterior mediastinum; **d** A video-assisted thoracoscopy had showed a smooth and nonulcerated nodular lesion(arrow) immediately below the normal mucosa

To our attention, the bone turnover makers, such as osteocalcin and I-type collagen prolongation peptide, had surpassed the upper normal limit(300 ng/ml and 1200 ng/ml, respectively), while the β-crosslaps was 5.96 ng/ml. In addition, the high bone turnover and remodeling rate, as well as the refractory HPT, had pointed to the presence of an ectopic parathyroid gland. Thus, the patient was further examined through computed tomography (CT). As expected, a contrast-enhanced neck CT scan had revealed a 2.5*1.9 cm mass in the upper region of anterior mediastinum, which had displayed nodular mixed density (Fig. 1a, b). More importantly, the Tc-99 m dual-phases parathyroid scan had detected a hyperfunctioning parathyroid gland in the same region(Fig. 1c). Consequently, it was concluded that the mass in the anterior mediastinum might be the ectopic parathyroid gland.

Despite of high anesthesia risk caused by hypocalcaemia, thoracic aortic calcification, and adherence to aortic arch, the mass was completely excised through video-assisted thoracoscopy (Fig. 1d). Biopsy was performed, and histopathological examination revealed a 2 cm well-encapsulated mass containing solid and cystic components. In addition, clear and transplant liquid could be observed inside the cyst, and a gray solid nodule had adhere to the wall. Microscope observation suggested that, the tumor was composed of the parathyroid gland chief cells, especially in the nodule.

Therefore, the calcium and PTH level had dropped stably within 10 days after surgery(2.12 mmol/l and 21.41 pmol/l, respectively), and they had been maintained for 3 months after surgery (2.30 mmol/l and 18.63 pmol/l, respectively). Besides, the phosphorus level(1.77 mmol/l) has dropped strikingly, while ALP bounced to 347u/l. The patient was then free from the restless legs syndrome and gastrointestinal distress. So far, he has been followed up for 16 months, and complications, including hungry bone syndrome, hypocalcemia, hemorrhage and infection, are not observed. On February 14th in 2019, the calcium, phosphorus, ALP and PTH were retested to be 2.42 mmol/l, 1.83 mmol/l, 70u/l and 13.77 pmol/l, respectively. Bone turnover had returned to normal level, since the levels of osteocalcin(121.3 ng/ml), I-type collagen prolongation peptide(213.5 ng/ml) and β-crosslaps (0.707 ng/ml) were reduced.

## Discussion and conclusions

Hypercalcemic crisis is currently defined as a syndrome with severe signs and symptoms that are reversible with hypercalcemia correction, and it is characterized by a serum calcium level over 3.5 mmol/l. When the parathyroid gland no longer responds to the serum calcium and secrete autonomously, it is often enlarged with focal or diffuse nodularity [3]. In this case, tertiary HPT had finally developed with persistent hypercalcemia, elevated serum PTH and an increase of the set-point calcium-PTH [1].

The treatment for tertiary HPT has post great challenges to both internists and surgeons. Although HPT caused by parathyroid adenoma was common, this case was novel for several reasons.

Firstly, the patient had undergone hemodialysis for end-stage renal disease. Unlike ordinary hypercalcemia, there was no obvious dehydration related to polyuria and polydipsia. As a result, fluid replacement or diuretic seemed to be insufficient. Accounting on calcium ions’ strong affinity to albumin, hemoperfusion and hemodiafiltration dialysate were useless.

Secondly, the patient has no response to medical therapy. Specifically, hasty application of active vitamin D without controlled phosphorus is not only ineffective, but might also aggravates the existing hypercalcemia and hyperphosphatemia [1]. Vitamin D analogs would increase calcium and phosphate absorption and thereby lead to hyperphosphatemia; therefore, the prime treatment of secondary HPT should be phosphate restriction [2]. Moreover, bone turnover makers should also be closely monitored after active vitamin D and their analogues were prescribed.

Calcimimetic agents can target CaSR, which play vital roles in regulating function of the parathyroid gland function. Typically, calcimimetic agents have better effects on secondary HPT patients with hypocalcemia, but they are rarely investigated in tertiary HPT [4]. It is suggested in one study that, calcimimetic agents are associated with the markedly increased ratio of oxyphil cells- chief cell ratio in the parathyroid gland [5]. On the other hand, cinacalcet would reduce the set-point of the PTH-Calcium curve [6], and is greatly beneficial for preventing PTx and hypercalcemia.

Thirdly, an ectopic parathyroid gland located in the anterior mediastinum was identified as the culprit of hypercalcemia and tertiary HPT in this case. However, the localization of the ectopic parathyroid glands vary diversely, and the most common sites are in the retroesophageal and thymic regions [7]. Notably, scintigraphy with 99mTc-Sestamibi should be performed in cases of recurrence and persistence of hyperparathyroidism. It does not reveal a simple parathyroid enlargement, instead, it can identify the presence of autonomous parathyroid issue that may indicate tertiary HPT [8] .

Fourthly, we also interested in why there was no enlarged parathyroid gland in the neck. Though the parathyroid gland embryologically originates from the endoderm tissue, the two superior glands descend differently from the two ones below [9]. The inferior glands which originate from the third pharyngeal pouch could descend along with the thymus, and be found in the neck or mediastinum [9]. It may be the differences in tissue origin that lead to their variable responses to pharmacological treatment.

Fifthly, unlike PTx for primary HPT, the indication for tertiary HPT is specially different. HPT alone with no other findings is not an indication for surgery, unless the increased levels of serum calcium are also detected [3]. PTx should be performed in patients who have failed the medical management [10, 11].

Finally, it is more difficult in the anesthetic management of the patient with tertiary HPT and end-stage renal disease, owing to uncontrolled hypercalcemia and poor capacity management. During anesthesia induction, arterial hypotension may develop at a remarkably higher frequency, along with the more ventricular premature beats [12]. Therefore, effective cooperation and communication among the anesthesia, surgical, endocrinology and nephrology staff are required to ensure the successful management of such patients [13].

After intervention, ongoing surveillance is also required. The high calcium level is the main driving force underlying the numerous symptoms and clinical manifestations in tertiary HPT patients [14]. Therefore, the goal of surgery for tertiary HPT is not to attain a normal PTH value, but to achieve a normal calcium level instead. It seems that the most appropriate level of postoperative iPTH is 21–150 pg/mL [15].

In summary, this study has reported a patient under hemodialysis who developed hypercalcemia and failed to be treated by internal medicine. Although no positive result is detected by ultrasound, radionuclide scanning can help us to find the presence of an ectopic parathyroid gland. The patient has a good prognosis after surgical resection. Taken together, our findings suggest that, it is of great significance to formulate multidisciplinary therapy on account of refractory hypercalcemia and HPT. Nonetheless, there are more to be explore.

## Abbreviations

ALP: Alkaline phosphatase
CaSR: Calcium-sensing receptor
CRRT: Continuous renal replacement therapy
CT: Computed tomography
HPT: Hyperparathyroidism
PTH: Parathyroid hormone
PTx: Parathyroidectomy
